# Whole Genome Sequencing Analysis of Model Organisms Elucidates the Association Between Environmental Factors and Human Cancer Development

**DOI:** 10.3390/ijms252011191

**Published:** 2024-10-17

**Authors:** Shinya Hasegawa, Yutaka Shoji, Mamoru Kato, Asmaa Elzawahry, Momoko Nagai, Min Gi, Shugo Suzuki, Hideki Wanibuchi, Sachiyo Mimaki, Katsuya Tsuchihara, Yukari Totsuka

**Affiliations:** 1 Department of Environmental Health Sciences, Hoshi University, 2-4-41 Ebara, Shinagawa-ku, Tokyo 142-8501, Japan; s-hasegawa@hoshi.ac.jp; 2 Department of Food Science and Nutrition, Shizuoka Eiwa Gakuin University Junior College, 1769 Ikeda, Suruga-ku, Shizuoka 422-8545, Japan; y.shoji@shizuoka-eiwa.ac.jp; 3 Division of Bioinformatics, National Cancer Center Research Institute, 5-1-1 Tsukiji, Chuo-ku, Tokyo 104-0045, Japan; mamkato@ncc.go.jp (M.K.); easmahgc@gmail.com (A.E.); monagai@ncc.go.jp (M.N.); 4 Department of Molecular Pathology, Osaka Metropolitan University Graduate School of Medicine, 1-4-3 Asahi-machi, Abeno-ku, Osaka 545-8585, Japan; o21773j@omu.ac.jp (M.G.); shugo@omu.ac.jp (S.S.); wani@omu.ac.jp (H.W.); 5 Division of Translational Informatics, Exploratory Oncology Research and Clinical Trial Center, National Cancer Center, 6-5-1 Kashiwanoha, Kashiwa, Chiba 277-8577, Japan; smimaki@east.ncc.go.jp (S.M.); ktsuchih@east.ncc.go.jp (K.T.)

**Keywords:** mutational signature, *N*-nitroso bile acid conjugates, 1,2-dichloropropane, occupational cholangiocarcinoma, inducible nitric oxide synthase

## Abstract

Determining a novel etiology and mechanism of human cancer requires extraction of characteristic mutational signatures derived from chemical substances. This study explored the mutational signatures of *N*-nitroso bile acid conjugates using *Salmonella* strains. Exposing *S. typhimurium* TA1535 to *N*-nitroso-glycine/taurine bile acid conjugates induced a predominance of C:G to T:A transitions. Two mutational signatures, B1 and B2, were extracted. Signature B1 is associated with *N*-nitroso-glycine bile acid conjugates, while Signature B2 is linked to *N*-nitroso-taurine bile acid conjugates. Signature B1 revealed a strong transcribed strand bias with GCC and GCT contexts, and the mutation pattern of *N*-nitroso-glycine bile acid conjugates in YG7108, which lacks *O*^6^-methylguanine DNA methyltransferases, matched that of the wild-type strain TA1535, suggesting that *O*^6^-methyl-deoxyguanosine contributes to mutations in the relevant regions. COSMIC database-based similarity analysis revealed that Signature B1 closely resembled SBS42, which is associated with occupational cholangiocarcinoma caused by overexposure to 1,2-dichlolopropane (1,2-DCP) and/or dichloromethane (DCM). Moreover, the inflammatory response pathway was induced by 1,2-DCP exposure in a human cholangiocyte cell line, and iNOS expression was positive in occupational cholangiocarcinomas. These results suggest that 1,2-DCP triggers an inflammatory response in biliary epithelial cells by upregulating iNOS and *N*-nitroso-glycine bile acid conjugate production, resulting in cholangiocarcinoma via DNA adduct formation.

## 1. Introduction

Mutations accumulate with age and multiple exposures, engraving characteristic mutational patterns or imprints in the genomes of somatic cells [1]. These patterns, termed mutational signatures, are classified according to the sequence surrounding the mutation sites. Approximately 100 types of mutational signatures have been reported in humans [2,3]. Mutational signatures reveal the underlying causes of mutations and reflect the activities of endogenous and exogenous mutational processes, such as life history and tumor etiology at the DNA level. Consequently, analyzing these signatures facilitates advancements in early cancer detection, prevention, and therapeutic strategies.

Notably, environmental factors primarily cause cancer rather than genetic factors [4]. While environmental influences such as dietary habits, smoking, and chemical exposures markedly contribute to cancer development, chemical substance-induced DNA mutations are major contributors to carcinogenesis. Approximately 70% of cancer cases are linked to environmental factors, with specific mutational signatures pointing to these causes [4]. However, many of these cases still lack known direct environmental triggers, underscoring the complexity of cancer etiology and highlighting the need for further investigation into unidentified carcinogenic factors. Accumulating data on the mutational signatures of chemical substances can aid in identifying cancer causes and elucidating the mechanisms of environmental carcinogenesis, with potential applications in cancer prevention research. Based on this idea, several mutational signature analyses of chemical substances using microorganisms, experimental animals, or mammalian cells have been reported, but most of them focus on mutagenic and carcinogenic substances present in the environment [5,6]. However, carcinogens are not only present in the ex vivo environment, but are also produced in our bodies. For example, patients undergoing distal gastrectomy have been reported to be at increased risk of gastric carcinoma, particularly after Billroth II resection, resulting in duodenogastric reflux [7]. In addition, Barrett’s esophagus, a major risk factor for esophageal adenocarcinoma, could be associated with prolonged reflux of the stomach and duodenal contents [8]. Indeed, in rats subjected to surgery to induce duodenal reflux into the stomach or esophagus, a high incidence of adenocarcinomas was observed in the stomach and esophagus [9,10]. Refluxed bile acids appear to markedly contribute to cancer development under these conditions.

Generally, bile acids are biosynthesized from cholesterol in the liver, mostly conjugated with glycine and taurine. They are metabolized by intestinal microflora in the gut and convert to secondary bile acids, which are then returned to the liver by intestinal circulation. Although bile acids serve various physiological functions, they have been known as promotors of carcinogenesis in various tissues, such as the colorectal and liver tissues, but bile acids themselves do not possess carcinogenic activity [11,12,13]. However, as described above, refluxed bile acids seem to contribute markedly to cancer development. Under reflux conditions, the acid-catalyzed reaction of amides with nitrite can mediate bile acid conjugate nitrosation [14], with activated macrophages in inflamed organs likely to be involved in such reactions. The *N*-nitrosation of bile acid conjugates, such as *N*-nitroso-glycocholic acid (NO-GCA) and *N*-nitroso-taurocholic acid (NO-TCA), exerts mutagenic activity in both bacterial and mammalian assay systems [14,15]. Moreover, these compounds induced liver, bile duct, and stomach cancers in F344 rats [16]. Some reports, including ours, document DNA adduct formation by these N-nitroso bile acid conjugates, and *O*^6^-methyl-deoxyguanosine (*O*^6^-Me-dG), *O*^6^-carboxymethyldeoxyguanosine (*O*^6^-CM-dG), and 3-ethanesulfonic acid-2′-deoxycytidine (3-ESA-dC) are the major products of NO-GCA or NO-TCA in vitro and in vivo [17,18,19]. These findings suggest that the synthesis of DNA adducts by *N*-nitroso bile acids is a key initiator of carcinogenesis, with *N*-nitrosation markedly contributing to many carcinogenic processes.

As humans are continuously exposed to bile acid conjugates, to clarify whether these endogenously produced carcinogens, such as *N*-nitroso bile acid conjugates, contribute to human cancer development, we used whole genome sequencing analysis to analyze their mutational signatures. Ames *Salmonella* strains were used as model organisms to analyze mutational signatures because we have already reported the mutagenicity of *N*-nitroso bile acid conjugates [18]. Two signatures were identified: one from the *N*-nitroso-glycine-bile acid conjugate and one from the *N*-nitroso-taurine bile acid conjugate. Of these, the signature related to the former was found to be quite similar to the mutational signature associated with occupational cholangiocarcinoma (SBS42), which we have already reported [20], suggesting that the *N*-nitrosation of bile acid conjugates is a critical factor in occupational cholangiocarcinoma development due to overexposure to haloalkanes, such as 1,2-dichloropropane (1,2-DCP) and dichloromethane (DCM). To further investigate the relationship between occupational cholangiocarcinoma and *N*-nitroso bile acid conjugates, RNA-seq analysis was performed using MMNK-1 cells, a highly differentiated immortalized human cholangiocyte cell line.

## 2. Results

### 2.1. Mutagenic Activity of N-Nitroso Bile Acid Conjugates in Salmonella Strains

**Figure 1:**
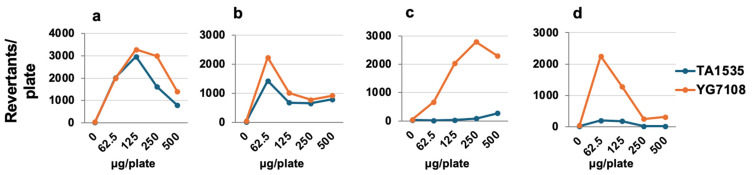
Mutagenicity of four types of *N*-nitroso bile acid conjugates, NO-TCA (**a**), NO-TDCA (**b**), NO-GCA (**c**), and NO-GDCA (**d**) in *Salmonella typhimurium* TA1535 and YG7108 without metabolic activation systems. Mutagenic activities observed in the wild-type TA1535 (blue) and YG7108 (orange), which lack *O*^6^-methylguanine DNA methyltransferases (MGMT), were estimated using the number of revertant colonies.

The mutagenic responses of four types of *N*-nitroso bile acid conjugates (NO-TCA, NO-TDCA, NO-GCA, and NO-GDCA) were evaluated in *Salmonella* strains TA1535 and YG7108 without metabolic activation. All conjugates showed mutagenicity to TA1535, but the mutagenic potency was higher in taurine conjugates than in the glycine conjugates (Figure 1). Previous research has shown that DNA adducts, such as *O*^6^-Me-dG and *O*^6^-CM-dG for glycine conjugates, and 3-ESA-dC and *N*^6^-cholyl-nucleic acid adducts, contribute to *N*-nitroso bile acid conjugate-induced mutagenic activity [18]. To confirm the effect of DNA repair enzyme deficiency on their mutagenic activities, we examined the mutation assay of those four types of *N*-nitroso bile acid conjugates with YG7108, which lacks *O*^6^-methylguanine DNA methyltransferases (MGMT) [21]. The YG7108 strain showed markedly higher *N*-nitroso-glycine conjugate-induced mutagenic activities than the wild-type TA1535, but *N*-nitroso-taurine conjugate-induced mutagenic activities were almost comparable in both *S. typhimurium* strains (Figure 1).

### 2.2. Analysis of DNA Adduct Formation

**Figure 2:**
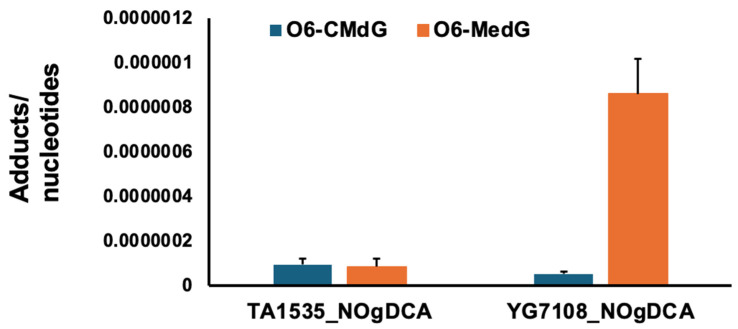
Levels of alkylating DNA adducts in TA1535 and YG7108 after incubation with NO-GDCA.

It appears that the *O*^6^-Me-dG and *O*^6^-CM-dG adducts formed by *N*-nitroso-glycine bile acid conjugates could not be repaired in YG7108, resulting in markedly elevated mutagenicity. To confirm this hypothesis, DNA adduct formation was analyzed in both MGMT-proficient and -deficient strains treated with NO-GDCA. The adduct levels of *O*^6^-Me-dG were significantly increased in YG7108, but not with *O*^6^-CM-dG (Figure 2).

### 2.3. Whole Genome Sequencing (WGS) of N-Nitroso-Glycine/Taurine Bile Acid Conjugate-Treated Salmonella Strains

**Figure 3:**
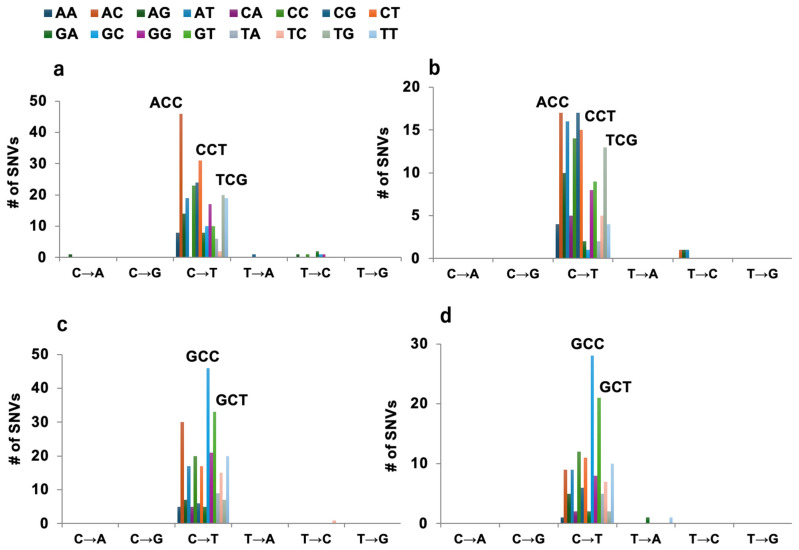
Trinucleotide mutational patterns for four types of *N*-nitroso bile acid conjugates, NO-TCA (**a**), NO-TDCA (**b**), NO-GCA (**c**), and NO-GDCA (**d**) in TA1535. The 96-pattern of mutational types are indicated by different colors. The vertical axis indicates the number of mutations. For *N*-nitroso-taurine conjugates, ACC, CCT, and TCG contexts were predominant, whereas for *N*-nitroso-glycine conjugates, GCC and GCT contexts were predominant.

To intensively analyze the effect of *N*-nitroso-glycine/taurine bile acid conjugates on DNA mutations, genomic DNA was extracted from TA1535 treated with these conjugates, and then subjected to WGS. Eight to ten clones from each treatment were analyzed. All clones, except one control (control 7), revealed mutations at positions 205 or 206 of the *hisG* gene, which is the target region of reversion mutations (Supplementary Table S1). C to T (or G to A in the complementary sequence) transitions were dominant (approximately 85%), followed by C to A (or G to T, 8.3%) and C to G (or G to C, 4.2%) transversions. While mutations other than the *hisG* gene were detected in most clones treated with *N*-nitroso bile acid conjugates, only two mutations were detected in the control clones. Table 1 lists the mutational patterns observed in TA1535 with and without *N*-nitroso bile acid conjugates. G:C to A:T transitions were dominant as the same as the *hisG* gene. Figure 3 shows the trinucleotide mutational pattern induced by each of the *N*-nitroso bile acid conjugates. As described, all of these *N*-nitroso bile acid conjugates predominantly induced C:G to T:A transitions. However, the mutational patterns of C:G to T:A transitions differ markedly between *N*-nitroso-taurine conjugates and *N*-nitroso-glycine conjugates, regardless of the chemical structures of bile acids (cholic acid vs. deoxycholic acid). While *N*-nitroso-taurine conjugates had ACC, CCT, and TCG contexts as predominant, *N*-nitroso-glycine conjugates had GCC and GCT contexts as predominant. Moreover, the trinucleotide mutational pattern of NO-GDCA remained unaffected by the MGMT status, whereas the number of SNVs was increased approximately three times in the MGMT-deficient strain, YG7108 (TA1535; 14 SNVs/clone vs. YG7108; 44 SNVs/clone) (Supplementary Figure S1).

**Figure 4:**
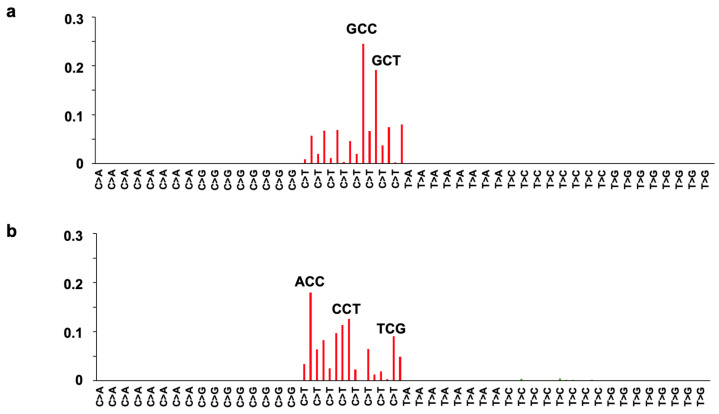
Extraction of mutational signatures from the 48 samples of *S. typhimurium* TA1535 exposed to *N*-nitroso bile acid conjugates by NMF analysis. Two mutational signatures, Signature B1 (**a**) and Signature B2 (**b**), were identified. The mutational signatures were normalized using the trinucleotide frequency in the genome. The horizontal axis represents the 96-pattern of mutational types in the same order as in Figure 3, and the vertical axis indicates the percentage of mutations attributed to a specific signature. Signature B1 features a unique trinucleotide mutational context of GCC and GCT, whereas Signature B2 has a prominent trinucleotide context of ACC, CCT, and TCG.

By using these genome data, the mutational signatures of the base substitutions by incorporating information regarding the 5′ and 3′ neighboring sites of each mutated base were extracted according to the procedure described in the Section 4. Two mutational signatures (Signature B1 and B2) with dominant mutations at the C to T transition were extracted (Figure 4). Supplementary Figure S2 shows the relative contribution of Signatures B1 and B2. Signature B1 features a unique trinucleotide mutational context of GCY to GTY, showing a major contribution among most clones treated with *N*-nitroso-glycine bile acid conjugates. In contrast, Signature B2, with prominent trinucleotide contexts of ACC, CCT, and TCG, showed a high contribution in the clones treated with *N*-nitroso-taurine bile acid conjugates.

**Figure 5:**
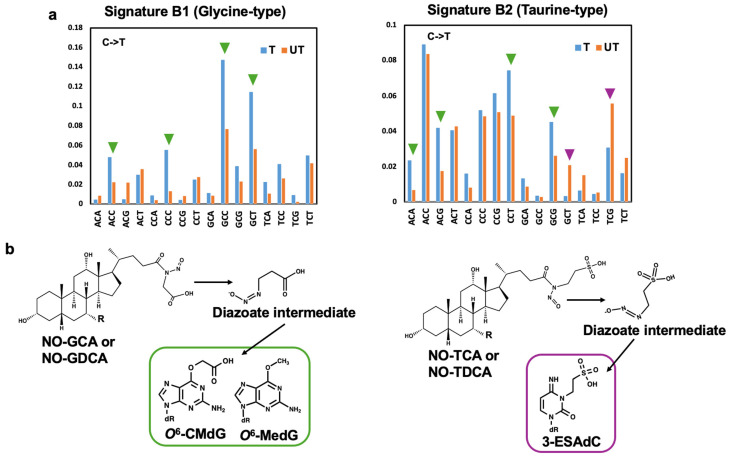
Strand bias pattern of the C to T transition in Signature B1/B2 (**a**). Green triangles indicate a strong strand bias on a transcribed strand, and purple triangles indicate that on an untranscribed strand. Formation of candidate DNA adducts that contribute to inducing a strand bias from bile acid conjugates (**b**).

Transcriptional strand bias was observed in the C to T mutations of Signatures B1 and B2 (Figure 5). For Signature B1, strong transcribed strand biases with several contexts, including GCC and GCT, were observed. In contrast, Signature B2 exhibited strong strand biases on the transcribed and untranscribed strands.

### 2.4. Comparison of Nitroso Bile Acid Conjugate Signatures Using the Catalog of Somatic Mutations in Cancer (COSMIC) Database

**Figure 6:**
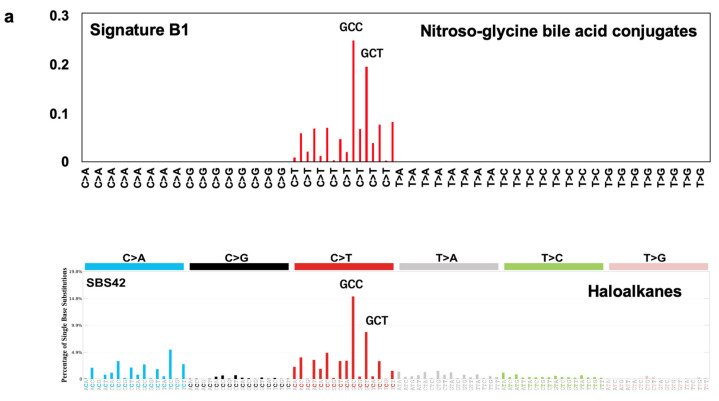

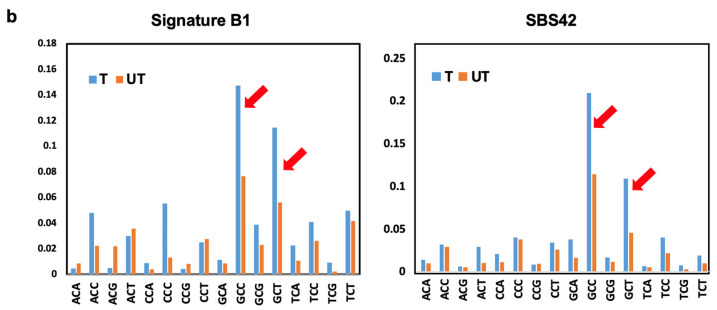
Comparison of Signature B1 and SBS42, a mutational signature of occupational cholangiocarcinoma caused by overexposure to 1,2-DCP (**a**). Significant strand bias for the C:G to T:A mutations were observed in the transcribed (T, blue) and untranscribed (UT, orange) strands in both Signature B1 and SBS42 (**b**). Red arrows indicated that similar transcriptional strand biases patterns.

Cosine similarity analysis with mutational signatures from the COSMIC database revealed that Signature B2, related to nitroso-taurine bile acid conjugates, is unique. In contrast, Signature B1, related to nitroso-glycine bile acid conjugates, is similar to SBS11 (cosine similarity, 0.76), SBS23 (0.78), SBS42 (0.86), and SBS84 (0.81) (Supplementary Table S2). In particular, the C to T mutational pattern of Signature B1 was almost identical to that of SBS42, a mutational signature of occupational cholangiocarcinoma caused by overexposure to a haloalkane (Figure 6a). Mutational signatures are widely recognized as evidence of environmental exposure to carcinogenesis. In fact, a strong renal carcinogen, aristolochic acid (AA), exhibits a unique A:T to T:A mutational signature that has been observed in numerous cases of renal cancer in populations from regions with high AA exposure [22]. Moreover, a strong strand bias between transcribed and nontranscribed strands in mutational signatures could further help identify and specify environmental exposure. Herein, both SBS42 and Signature B1 revealed strong transcriptional strand biases, and these patterns were also quite similar (Figure 6b) [20]. These results indicate that *N*-nitroso-glycine bile acid conjugates may be involved in haloalkane-induced occupational cholangiocarcinoma development.

### 2.5. 1,2-DCP Treatment Induces Inflammatory Genes in Cholangiocyte Cells

**Figure 7:**
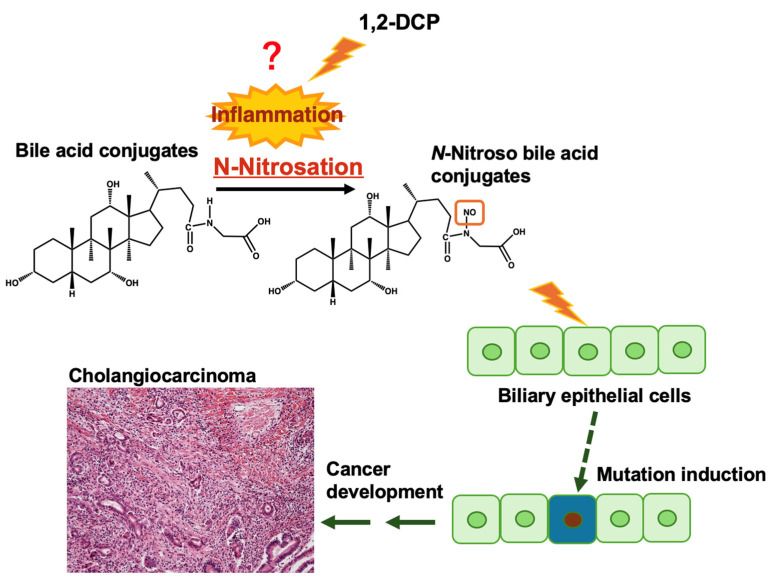
Possible mechanisms of 1,2-DCP associated with occupational cholangiocarcinoma. Excessive exposure to 1,2-DCP could induce inflammation and mediate the *N*-nitrosation of glycine bile acid conjugates. These *N*-nitroso-glycine bile acid conjugates likely affect biliary epithelial cells, ultimately inducing occupational cholangiocarcinoma.

Based on the mutational signature data, we hypothesized that nitroso-glycine bile acid conjugates could be mediated by an excess amount of haloalkane exposure. These *N*-nitroso-glycine bile acid conjugates likely affect biliary epithelial cells, ultimately inducing occupational cholangiocarcinoma (Figure 7). A previous report suggested that 1,2-DCP is a more suspicious causative agent than DCM because three of the four printing workers’ cholangiocarcinoma cases were exposed only to 1,2-DCP and not to DCM [20]. To confirm this hypothesis, we used RNA sequencing analysis to investigate the effect of 1,2-DCP on a highly differentiated immortalized human cholangiocyte cell line (MMNK-1 cells). After 4 h of treatment of MMNK-1 cells with 1,2-DCP (20 μL/plate; 1200 ppm), total RNA was isolated and subjected to RNA sequencing. Table 2 lists the upregulated pathways identified using the MSigDB Hallmark 2020 pathway analysis. The data were analyzed to identify significant pathways, and the top 10 pathways were sorted by a combined score. The top pathway was “TNF-alpha signaling via NF-kB” with a combined score of 541.19. Additionally, “Inflammatory Response” was identified with a combined score of 11.37. These results suggest that the inflammatory response pathway was induced in the early stages of 1,2-DCP exposure.

### 2.6. iNOS Expression in Occupational Cholangiocarcinomas

**Figure 8:**
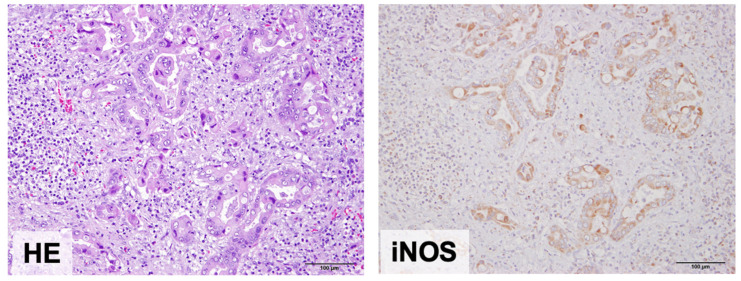
Expression of iNOS in occupational cholangiocarcinoma. Representative data of immunohistochemical staining for iNOS from occupational cholangiocarcinoma is shown. iNOS staining was localized to the cytoplasm of inflammatory and cancer cells and tended to be overexpressed for cancer cells in areas with significant inflammatory cell infiltration. Bars are 100 μm.

To confirm the hypothesis that iNOS induction, which produces nitrate as part of the inflammatory response, could be observed in occupational cholangiocarcinoma patients, we investigated iNOS expression in occupational cholangiocarcinoma using immunohistochemical analysis. iNOS staining was localized to the cytoplasm of inflammatory and cancer cells but negative in the normal bile duct epithelial cells in the paired non-tumor liver tissues (Figure 8). iNOS was overexpressed in all three occupational cholangiocarcinomas. Cancer cells in areas with significant inflammatory cell infiltration tended to overexpress iNOS. Furthermore, *iNOS* expression was significantly induced via 1,2-DCP treatment in MMNK-1 cells (Supplementary Figure S3). These results suggest that nitric oxide synthesized by iNOS, induced by the inflammatory response to 1,2-DCP overexposure, is associated with the synthesis of *N*-nitroso-glycine bile acid conjugates.

## 3. Discussion

The present study examined the mutagenicity of NO-TCA, NO-TDCA, NO-GCA, and NO-GDCA in TA1535. Results indicated that *N*-nitroso-taurine bile acid conjugates exhibited markedly higher mutagenic potency than *N*-nitroso-glycine-bile acid conjugates. This difference seems to be linked more to the type of amino acid conjugation rather than the bile acid itself. Particularly, nitroso-glycine conjugates form simple alkylating adducts, such as *O*^6^-Me-dG and *O*^6^-CM-dG, whereas nitroso-taurine conjugates produce bulky adducts, such as 3-ESA-dC and *N*^6^-cholyl-dC/dA adducts, which could contribute to their higher mutagenic potency than alkylating adducts. While YG7108, which lacks MGMT, exhibited markedly higher *N*-nitroso-glycine conjugate-induced mutagenic activities than the wild-type TA1535, *N*-nitroso-taurine conjugate-triggered mutagenic activities were almost comparable in both *S. typhimurium* strains. As nucleotide excision repair mechanisms repair bulky adducts such as *N*^6^-cholyl-dC/dA [23], these bulky adducts and 3-ESA-dC may be repaired using mechanisms other than MGMT. Comparison results of DNA adduct formations in TA1535 and YG7108, whereby *O*^6^-Me-dG levels were dramatically elevated but *O*^6^-CM-dG levels were not, suggest that the induction of mutagenicity observed in YG7108 might be due to *O*^6^-Me-dG.

WGS of clones with reversion mutations revealed that exposure to *N*-nitroso bile acid conjugates led to numerous base substitutions outside the *hisG* gene, unlike control clones. Using these mutation data, NMF analysis yielded two mutational signatures: Signatures B1 and B2. The contribution of the signatures suggests that Signature B1 is associated with the *N*-nitroso-glycine bile acid conjugate and Signature B2 with the *N*-nitroso-taurine bile acid conjugate. Although both mutational signatures revealed that the C to T transition was dominant, their trinucleotide context pattern completely differed. This disparity likely occurs due to the different types of DNA adduct formations, as described above. Furthermore, Signature B1 may have resulted from the *O*^6^-Me-dG adduct as the trinucleotide mutation pattern of NO-GDCA was not affected by MGMT status and *O*^6^-Me-dG levels increased in MGMT-deficient strains. In addition, a considerable strand bias was observed in Signatures B1 and B2. Strand bias refers to the phenomenon in which DNA damage, such as adducts generated on the transcribed strand, is actively repaired by transcription-coupled repair but is less likely to be repaired on the nontranscribed strand. This results in different mutation frequencies on the transcribed and nontranscribed strands [24]. For Signature B1, a strong strand bias could be observed in GCC, GCT, and TCC contexts on the transcribed strand, suggesting guanine adduct involvement in these relevant region’s mutations. As mentioned above, *O*^6^-Me-dG may contribute to the G to A transition of these contextual mutations, and indeed, G to A mutations have been reported to be a feature of alkylating agents via *O*^6^-Me-dG production [25,26,27]. Similarly, *N*-nitroso-taurine bile acid conjugates are the etiology of Signature B2, and a strong strand bias was observed in the GCT and TCG contexts on the untranscribed strand—3-ESA-dC may have contributed to these context mutations [18]. A strong strand bias in the ACG, CCT, and GCG contexts on the transcribed strand is suggested to be involved in guanine adduct formation, but no information currently exists regarding guanine adduct formation by *N*-nitroso-taurine bile acid conjugates. However, at present, proving a direct relationship between DNA adducts and mutational signatures is difficult. A recent study explored the detection and identification of DNA adducts in the genome using an Oxford Nanopore Technology (nanopore sequencer) [28]. The sequencing principle of the nanopore sequencer is based on sensing changes in ionic currents when a single molecule of single-stranded DNA passes through a small pore; when a base modified with a chemical substance such as a DNA adduct passes through a small pore, it senses a different change in ionic current than a normal base; therefore, DNA adducts can be detected. Detecting 5-methyl-cytosine, known as epigenome, is already possible, and recent reports have also described the detection of 8-hydroxy-dG, oxidative damage to DNA [29,30]. Developing a method for detecting nitroso-glycine/taurine bile acid conjugate-related DNA adducts, such as *O*^6^-Me-dG or 3-ESA-dC, by nanopore sequencing is possible. Detecting these adducts in contexts with a strong strand bias of Signature B1/B2 could prove the association between these DNA adducts and mutational signatures.

Similarity analysis reveals that Signature B1 shares a high rate of similarity with a mutational signature of occupational cholangiocarcinoma caused by overexposure to 1,2-DCP (SBS42). SBS42 revealed a strong transcriptional strand bias in the GCC and GCT contexts, similar to Signature B1. This suggests that *N*-nitroso-glycine bile acid conjugates may contribute to occupational cholangiocarcinoma development. Notably, NO-GCA induced hepatocellular carcinoma, gastric adenocarcinoma, and cholangioma in rats [16]. We have reported that 1,2-DCP exhibits a partially similar mutational signature to SBS42, but the mechanisms of occupational cholangiocarcinoma development remain unclarified [20]. Moreover, neither 1,2-DCP nor DCM, the chemicals responsible for occupational cholangiocarcinoma, induced bile duct cancer in experimental animals [31,32,33]. Thus, these chemicals may indirectly contribute to occupational cholangiocarcinoma development. In our previous paper, we described how massive fibrosis and infiltration of inflammatory cells were frequently observed in the histopathological analysis of patients with occupational cholangiocarcinoma [20]. This means that severe inflammation status was continuously observed in these patients. Thus, the generation of *N*-nitroso-glycine-bile acid conjugates may occur via nitric oxide production by iNOS expression induced by exposure to excessive amounts of 1,2-DCP under inflammatory conditions. This condition may continue for a certain period to form *O*^6^-Me-dG in bile duct epithelial cells and induce mutations. Supporting this hypothesis, the signaling pathways related to TNF-alpha signaling and inflammatory responses were elevated in the MMNK-1 cells treated with 1,2-DCP. In addition, *iNOS* mRNA levels were significantly upregulated in 1,2-DCP-treated MMNK-1 cells, and iNOS staining was observed for cancer cells in areas with significant inflammatory cell infiltration in occupational cholangiocarcinoma patients.

In conclusion, this study analyzed the mutational signatures of *N*-nitroso bile acid conjugates using *Salmonella* strains to determine the novel etiology of human cancer. Exposure of *S. typhimurium* TA1535 to *N*-nitroso-glycine/taurine bile acid conjugates predominantly caused C:G to T:A transitions. Notably, *N*-nitroso-glycine bile acid conjugates displayed a unique mutational signature with GCC and GCT contexts, showing a resemblance to the human mutational signature SBS42, which is associated with occupational cholangiocarcinoma from haloalkane exposure. This is the first report to describe that *N*-nitroso-glycine bile acid conjugates can form endogenously in the human body and contribute to cancer development. This finding also suggests that non-genotoxic/carcinogenic bile acids can be converted into genotoxic/carcinogenic compounds through processes driven by severe and prolonged inflammation, as observed in cases of occupational exposure. Regarding occupational carcinogenesis, the direct interaction between chemical substances and DNA is strongly thought to result in cancer development through several driver gene mutation inductions. However, the study findings suggest a novel mechanism for occupational cancer development. As bile acids are biosynthesized in the liver and humans cannot avoid exposure to these compounds, frequent endogenous *N*-nitrosation of bile acids in the body is unlikely. This conclusion is supported by the fact that SBS42 has been observed in only a limited number of occupational bile duct cancers. However, the possibility that other chemical exposures could induce similar phenomena cannot be ruled out. Even when exposure is an endogenous factor that is difficult to avoid, if the carcinogenic mechanism can be identified, preventive methods may be possible.

Despite the large amount of mutational signature information currently contained in databases, only a limited number of these etiologies are known. Elucidation of causal factors for human cancer is crucial for constructing cancer prevention strategies. Therefore, further research is needed to continue collecting information on mutational signatures derived from environmental factors using animal models, mammalian cells, and microorganisms. This work will identify poorly understood human carcinogenic factors and mechanisms.

## 4. Materials and Methods

### 4.1. Materials

NO-TCA, *N*-nitroso-taurodeoxycholic acid (NO-TDCA), NO-GCA, and *N*-nitroso-glycodeoxycholic acid (NO-GDCA) were obtained from the Nard Institute (Osaka, Japan). *O*^6^-Me-dG was purchased from USBiological Life Sciences (Salem, MA, USA), and *O*^6^-CM-dG was chemically synthesized using the same procedures previously reported [19].

### 4.2. Mutagenicity Assay

An Ames assay was conducted to test the mutagenicity of *N*-nitroso bile acid conjugates to *S. typhimurium* TA1535 and YG7108, based on a previously reported strategy [18]. Briefly, bacterial cells were incubated with the test chemical without S9 mix for 20 min at 37 °C in a total volume of 0.7 mL. The mixture was poured onto agar plates with 2 mL of soft agar and incubated for 2 days at 37 °C. The number of hisG+ revertants per plate was then determined.

### 4.3. WGS of Nitroso Bile Acid Conjugate-Treated Salmonella Strains

The hisG+ colonies were randomly isolated and subcultured before genomic DNA was extracted using a Puregene Cell and Tissue kit (Qiagen, Hilden, Germany). WGS was performed using the previously described procedure [34]. Briefly, 3 μg of DNA was used, and the NextraXT DNA Library Prep Kit (Agilent Technologies, Santa Clara, CA, USA) was used to prepare the WGS libraries according to the manufacturer’s protocol. The prepared libraries were sequenced using a MiSeq system (Illumina, San Diego, CA, USA) to generate 100 bp paired-end data. The reads were aligned to the reference genome NC_003197 (*Salmonella enterica* subsp. *enterica* serovar *typhimurium* strain LT2, complete genome, 4,857,432 bp) using the Burrows–Wheeler Aligner v. 07.18 (BWA, http://bio-bwa.sourceforge.net/, accessed on 9 October 2024). Single nucleotide variants (SNVs) were identified and annotated using the Genome Analysis Toolkit software package 4.beta.1 (GATK, http://www.broadinstitute.org/gatk/, accessed on 9 October 2024). We selected “PASS” variants annotated by the GATK program as high-confidence acquired variants.

### 4.4. Mutational Signature Analysis

We utilized non-negative matrix factorization (NMF) to identify the mutational signatures using the Wellcome Trust Sanger Institute’s framework [35]. We input the datasets of the 96-trinucleotide substitution frequencies from the 48 samples (Control: 10, NO-TCA: 10, NO-TDCA: 8, NO-GCA: 10, NO-GDCA: 10) for TA1535 and 60 samples (Control: 20, NO-GCA: 28, NO-GDCA: 12) for YG7108.

### 4.5. Comparison with COSMIC Mutational Signatures

Cosine similarity was used to compare the identified mutational signatures in bacteria, with approximately 100 human mutational signatures registered in the COSMIC database, v3.4. (http://cancer.sanger.ac.uk, accessed on 9 October 2024).

### 4.6. DNA Adduct Analysis

Overnight cultures of *S. typhimurium* TA1535 and YG7108 (~20 mL) were centrifuged, and the bacterial pellets were washed and resuspended in 2 mL of PBS. NO-GDCA, at a final concentration of 90 μg/mL, was incubated with these bacterial solutions for 6 h, after which DNA was extracted using the same procedures as described above and stored at −80 °C until further use. We prepared three independent samples for each group. The extracted DNA was digested with DNase I, nuclease P1, and alkaline phosphatase, as previously described [36]. The amounts of *O*^6^-Me-dG and *O*^6^-CM-dG were measured using LC-MS analysis performed with a Shimadzu Prominence LC system interfaced with a Triple TOF6600 mass spectrometer (SCIEX, Framingham, MA, USA) in the product ion (PI) mode. The HPLC conditions were as follows: a SynergiTM Fusion-RP column (2.5 mm particle size, 2.0 × 100 mm; Phenomenex, Torrance, CA, USA); flow rate 0.4 mL/min; and solvent system, a linear gradient from 2.5% to 85% acetonitrile in 10 mM ammonium acetate (pH 5.3) for 30 min. *O*^6^-Me-dG and *O*^6^-CM-dG were analyzed in PI mode, with major fragment ions *m*/*z* 282.11→166.07 corresponding to the loss of the deoxyribose moiety (*O*^6^-Me-G) and *m*/*z* 326.10→210.06 corresponding to the loss of the deoxyribose moiety (*O*^6^-CM-G), with a cone voltage of 35 V and collision energy of 20 eV. Quantification was based on a standard curve of authentic *O*^6^-Me-dG and *O*^6^-CM-dG, with a detection limit of 0.03 pmol/injection. Relative DNA adduct levels were calculated as adducts per normal nucleotide.

### 4.7. RNA-Seq Analysis

The highly differentiated, immortalized human cholangiocyte cell line MMNK-1 was purchased from the JCRB cell bank and maintained in DMEM medium (Nacalai Tesque INC, Kyoto, Japan) containing 5% fetal bovine serum. An in vitro vapor exposure system, which was previously reported, was used [20]. In brief, appropriately sized filter papers were placed into two rhombus-shaped holes in the center of a six-well culture plate, and varying doses of 1,2-DCP (10–40 μL/plate) were absorbed onto the filter papers. The chemical vapor concentrations in the plastic bags were calculated with calibration curves analyzed by gas chromatography–mass spectrometry, as described previously [20]. The culture plates were placed in sealed plastic bags. Using this method, 1,2-DCP evaporated and dissolved in the culture medium [37]. After 4 h of vapor exposure for MMNK-1, the cells were harvested, and total RNA was extracted using Trizol (Thermo Fisher, Waltham, MA, USA), followed by re-extraction with the RNeasy Mini Kit (QIAGEN), following the manufacturer’s procedures. RNA-seq analysis was performed at Takara Bio Inc. (Shiga, Kusatsu, Japan).

### 4.8. Real-Time PCR Analysis

Total RNA was isolated from the MMNK-1 cells with or without 1,2-DCP exposure using Trizol (Thermo Fisher Scientific). Aliquots (100 ng) in a final volume of 20 μL were used for cDNA synthesis using SuperScript IV VILO Master Mix (Thermo Fisher Scientific) and oligo primers. Real-time PCR was performed using ViiA7 (Thermo Fisher) with KAPA SYBR FAST qPCR Master Mix (2×) (KAPA BIOSYSTEMS) according to the manufacturer’s instructions. Primers for human inducible NO synthase (*iNOS*) (F: CAGCGGGATGACTTTCCAA and R: AGGCAAGATTTGGACCTGCA) and peptidylprolyl isomerase A (PPIA) (F: GGAGGCCAGGCTCGTGC and R: CTTGTCTGCAAACAGCTCA) were used.

### 4.9. Preparation of Clinical Samples

Samples from three male patients with occupational cholangiocarcinoma who underwent partial liver resection at Osaka Metropolitan University Hospital (formerly Osaka City University Hospital) in 2012 were analyzed. All patients worked at the same printing company and were exposed to 1,2-DCP and/or DCM. Cholangiocarcinoma specimens and paired non-tumor liver tissues were immunohistochemically analyzed for iNOS. The median age was 37 years (range, 31–47 years). The Institutional Review Board at Osaka Metropolitan University Graduate School of Medicine (Former Osaka City University Graduate School of Medicine) approved the use of the specimens and clinical data in accordance with the Declaration of Helsinki and the guidelines of the Osaka Metropolitan University Graduate School of Medicine.

### 4.10. Immunohistochemical Analysis

Serial sections (4 µm thickness) cut from paraffin-embedded occupational cholangiocarcinoma specimens were examined for inducible nitric oxide synthase (iNOS) expression by immunohistochemical staining using the avidin–biotin–peroxidase complex (ABC) method. Antigen retrieval was performed for rat sections by microwaving at 98 °C for 20 min in 0.01 M citrate buffer (pH 6.0). Endogenous peroxidase activity was blocked with 3% H_2_O_2_ in distilled water for 5 min. After blocking nonspecific binding with goat serum at 37 °C for 30 min, sections were incubated with mouse monoclonal anti-iNOS/NOS Type II (#610329, BD Transduction Laboratories, San Diego, CA, USA) diluted 1:50 overnight at 4 °C. Immunoreactivity was detected using a VECTASTAIN Elite ABC Kit (PK-6102, Vector Laboratories, Burlingame, CA, USA) and 3,3′-diaminobenzidine hydrochloride (Sigma Chemical Co., St. Louis, MO, USA). Omission of the primary antibody served as the negative control and was included in each staining procedure. Overexpression of iNOS in occupational cholangiocarcinoma was defined as positive when cytoplasmic staining was evident in >10% of the cancer cells.

## Figures and Tables

**Table 1:** Mutations detected in TA1535 treated with *N*-nitroso bile acid conjugates by whole genome sequencing (except for the *hisG* gene).

	Type of Mutation		Control (*n* = 10)	NO-TCA (*n* = 10) 180 mg/mL	NO-TDCA (*n* = 8) 90 mg/mL	NO-GCA (*n* = 10) 360 mg/mL	NO-GDCA (*n* = 10) 90 mg/mL
Base substitution	Transition	G:C to A:T	1	260	142	263	138
		A:T to G:C	0	7	3	1	0
	Transversion	G:C to T:A	0	1	0	0	0
		G:C to C:G	0	0	0	0	0
		A:T to T:A	0	1	0	0	0
		A:T to C:G	1	0	0	0	0
	Insertion		0	0	0	0	0
	Deletion		0	0	0	0	1
	Total		2	269	145	264	141

**Table 2:** Top 10 enriched pathways in the cholangiocyte cell line treated with 1,2-DCP using the MSigDB Hallmark 2020 process.

Index	Name	*p*-Value	Adjusted *p*-Value	Odds Ratio	Combined Score
1	TNF-alpha Signaling via NF-κB	1.61 × 10^−42^	7.26 × 10^−41^	19.08	1836.16
2	Hypoxia	1.04 × 10^−15^	2.33 × 10^−14^	8.28	285.51
3	TGF-beta Signaling	1.26 × 10^−07^	1.13 × 10^−6^	11.11	176.46
4	UV Response Up	2.81 × 10^−8^	3.72 × 10^−7^	5.97	103.75
5	p53 Pathway	3.31 × 10^−8^	3.72 × 10^−7^	5.21	89.75
6	Unfolded Protein Response	2.10 × 10^−5^	1.57 × 10^−4^	5.27	56.73
7	Apoptosis	1.25 × 10^−4^	6.26 × 10^−4^	3.93	35.35
8	Inflammatory Response	6.92 × 10^−5^	3.89 × 10^−4^	3.69	35.33
9	Estrogen Response Late	6.92 × 10^−5^	3.89 × 10^−4^	3.69	35.33
10	Hedgehog Signaling	6.11 × 10^−3^	1.72 × 10^−2^	6.02	30.70

## Data Availability

The data presented in this study are available in the article.

## Supplementary Materials

The following supporting information can be downloaded at: https://www.mdpi.com/article/10.3390/ijms252011191/s1.
