# Supplementary material for: Whole Genome Sequencing Analysis of Model Organisms Elucidates the Association Between Environmental Factors and Human Cancer Development
Source: Int J Mol Sci. 2024 Oct 17;25(20):11191. doi: 10.3390/ijms252011191 (PMC11508241; doi:10.3390/ijms252011191)
Supplement: Supplementary file 1 [file ijms-25-11191-s001.zip › ijms-3208693-supplementary/Supplementary figures.pptx]

## Slide 1
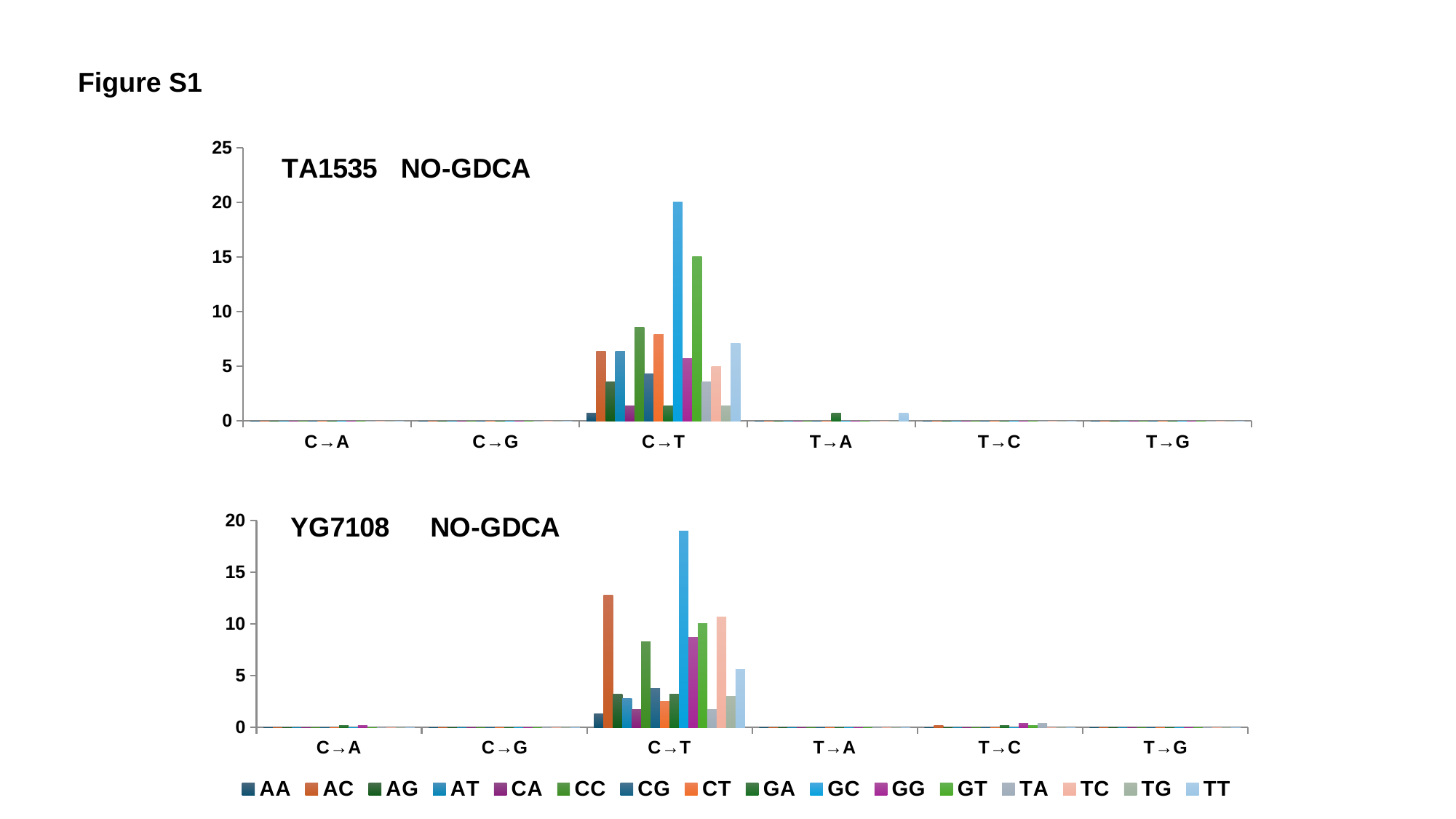

Figure S1
### Chart: TA1535 NO-GDCA
| Category | AA | AC | AG | AT | CA | CC | CG | CT | GA | GC | GG | GT | TA | TC | TG | TT |
|---|---|---|---|---|---|---|---|---|---|---|---|---|---|---|---|---|
| C→A | 0.0 | 0.0 | 0.0 | 0.0 | 0.0 | 0.0 | 0.0 | 0.0 | 0.0 | 0.0 | 0.0 | 0.0 | 0.0 | 0.0 | 0.0 | 0.0 |
| C→G | 0.0 | 0.0 | 0.0 | 0.0 | 0.0 | 0.0 | 0.0 | 0.0 | 0.0 | 0.0 | 0.0 | 0.0 | 0.0 | 0.0 | 0.0 | 0.0 |
| C→T | 0.7 | 6.4 | 3.6 | 6.4 | 1.4 | 8.6 | 4.3 | 7.9 | 1.4 | 20.0 | 5.7 | 15.0 | 3.6 | 5.0 | 1.4 | 7.1 |
| T→A | 0.0 | 0.0 | 0.0 | 0.0 | 0.0 | 0.0 | 0.0 | 0.0 | 0.7 | 0.0 | 0.0 | 0.0 | 0.0 | 0.0 | 0.0 | 0.7 |
| T→C | 0.0 | 0.0 | 0.0 | 0.0 | 0.0 | 0.0 | 0.0 | 0.0 | 0.0 | 0.0 | 0.0 | 0.0 | 0.0 | 0.0 | 0.0 | 0.0 |
| T→G | 0.0 | 0.0 | 0.0 | 0.0 | 0.0 | 0.0 | 0.0 | 0.0 | 0.0 | 0.0 | 0.0 | 0.0 | 0.0 | 0.0 | 0.0 | 0.0 |
### Chart: YG7108　NO-GDCA
| Category | AA | AC | AG | AT | CA | CC | CG | CT | GA | GC | GG | GT | TA | TC | TG | TT |
|---|---|---|---|---|---|---|---|---|---|---|---|---|---|---|---|---|
| C→A | 0.0 | 0.0 | 0.0 | 0.0 | 0.0 | 0.0 | 0.0 | 0.0 | 0.2 | 0.0 | 0.2 | 0.0 | 0.0 | 0.0 | 0.0 | 0.0 |
| C→G | 0.0 | 0.0 | 0.0 | 0.0 | 0.0 | 0.0 | 0.0 | 0.0 | 0.0 | 0.0 | 0.0 | 0.0 | 0.0 | 0.0 | 0.0 | 0.0 |
| C→T | 1.3 | 12.8 | 3.2 | 2.8 | 1.7 | 8.3 | 3.8 | 2.5 | 3.2 | 19.0 | 8.7 | 10.0 | 1.7 | 10.7 | 3.0 | 5.6 |
| T→A | 0.0 | 0.0 | 0.0 | 0.0 | 0.0 | 0.0 | 0.0 | 0.0 | 0.0 | 0.0 | 0.0 | 0.0 | 0.0 | 0.0 | 0.0 | 0.0 |
| T→C | 0.0 | 0.2 | 0.0 | 0.0 | 0.0 | 0.0 | 0.0 | 0.0 | 0.2 | 0.0 | 0.4 | 0.2 | 0.4 | 0.0 | 0.0 | 0.0 |
| T→G | 0.0 | 0.0 | 0.0 | 0.0 | 0.0 | 0.0 | 0.0 | 0.0 | 0.0 | 0.0 | 0.0 | 0.0 | 0.0 | 0.0 | 0.0 | 0.0 |

## Slide 2
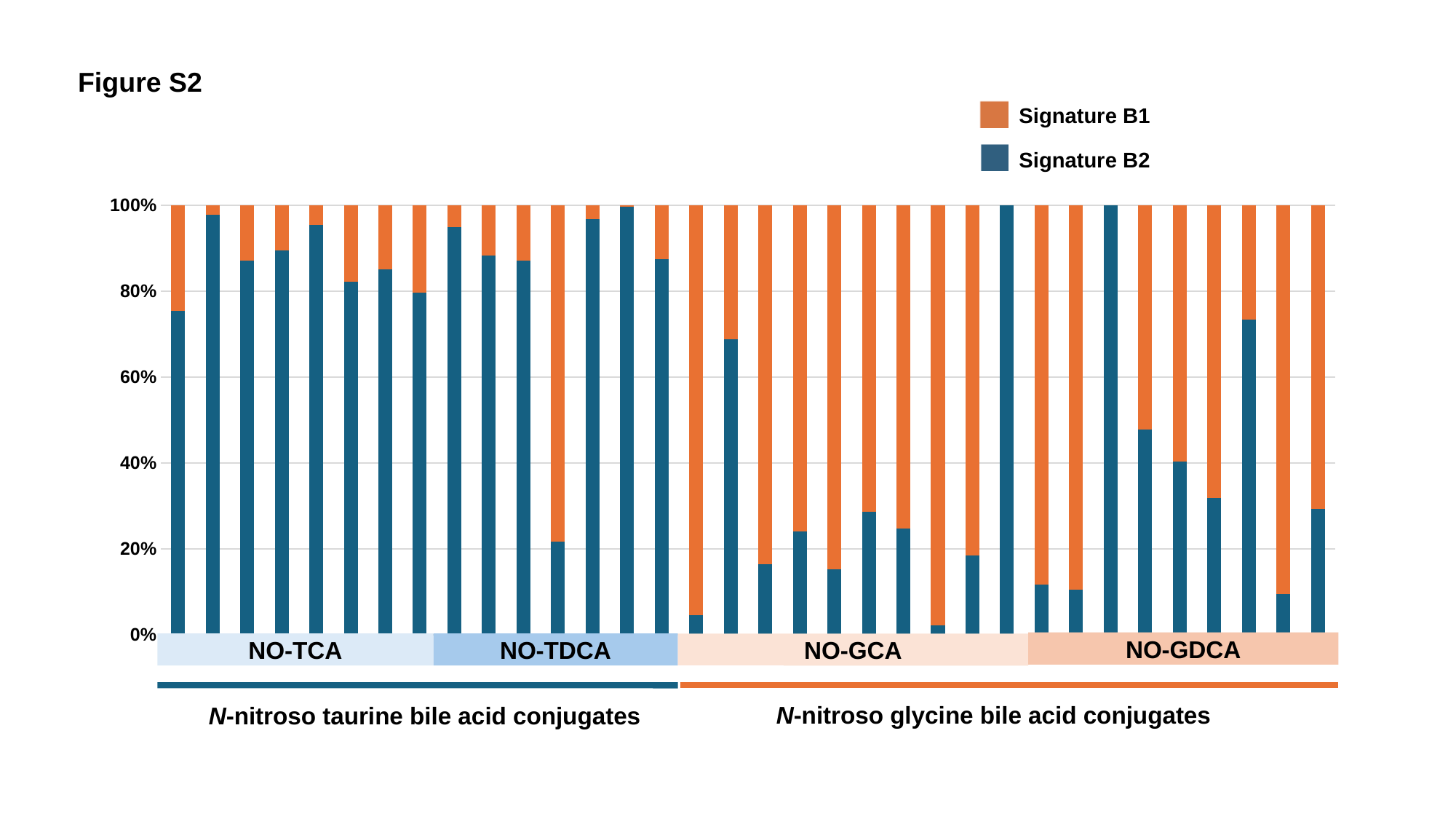

Figure S2
Signature B1
Signature B2
### Chart
| Category | Sig1 | Sig2 |
|---|---|---|
| A-10 | 50.6 | 16.4 |
| A-1 | 4.89 | 0.109 |
| A-2 | 72.4 | 10.6 |
| A-3 | 7.159999999999997 | 0.837 |
| A-4 | 15.3 | 0.716 |
| A-5 | 22.2 | 4.81 |
| A-7 | 36.6 | 6.43 |
| A-9 | 12.7 | 3.25 |
| B-1 | 13.3 | 0.712 |
| B-2 | 11.5 | 1.53 |
| B-3 | 32.30000000000001 | 4.74 |
| B-4 | 4.13 | 14.9 |
| B-5 | 38.80000000000001 | 1.25 |
| B-6 | 15.9 | 0.0618 |
| B-7 | 3.5 | 0.498 |
| C-10 | 1.01 | 21.0 |
| C-1 | 40.6 | 18.4 |
| C-2 | 2.31 | 11.7 |
| C-3 | 12.3 | 38.7 |
| C-4 | 3.5 | 19.5 |
| C-5 | 4.88 | 12.1 |
| C-6 | 13.1 | 39.9 |
| C-7 | 0.087 | 3.91 |
| C-8 | 3.72 | 16.3 |
| C-9 | 1.0 | 2.24e-15 |
| D-10 | 3.62 | 27.4 |
| D-1 | 1.57 | 13.4 |
| D-2 | 1.0 | 2.19e-15 |
| D-3 | 0.478 | 0.522 |
| D-4 | 11.7 | 17.3 |
| D-5 | 1.91 | 4.09 |
| D-7 | 1.47 | 0.531 |
| D-8 | 2.39 | 22.6 |
| D-9 | 8.239999999999998 | 19.8 |NO-GDCA
NO-TCA
NO-TDCA
NO-GCA
N-nitroso glycine bile acid conjugates
N-nitroso taurine bile acid conjugates

## Slide 3
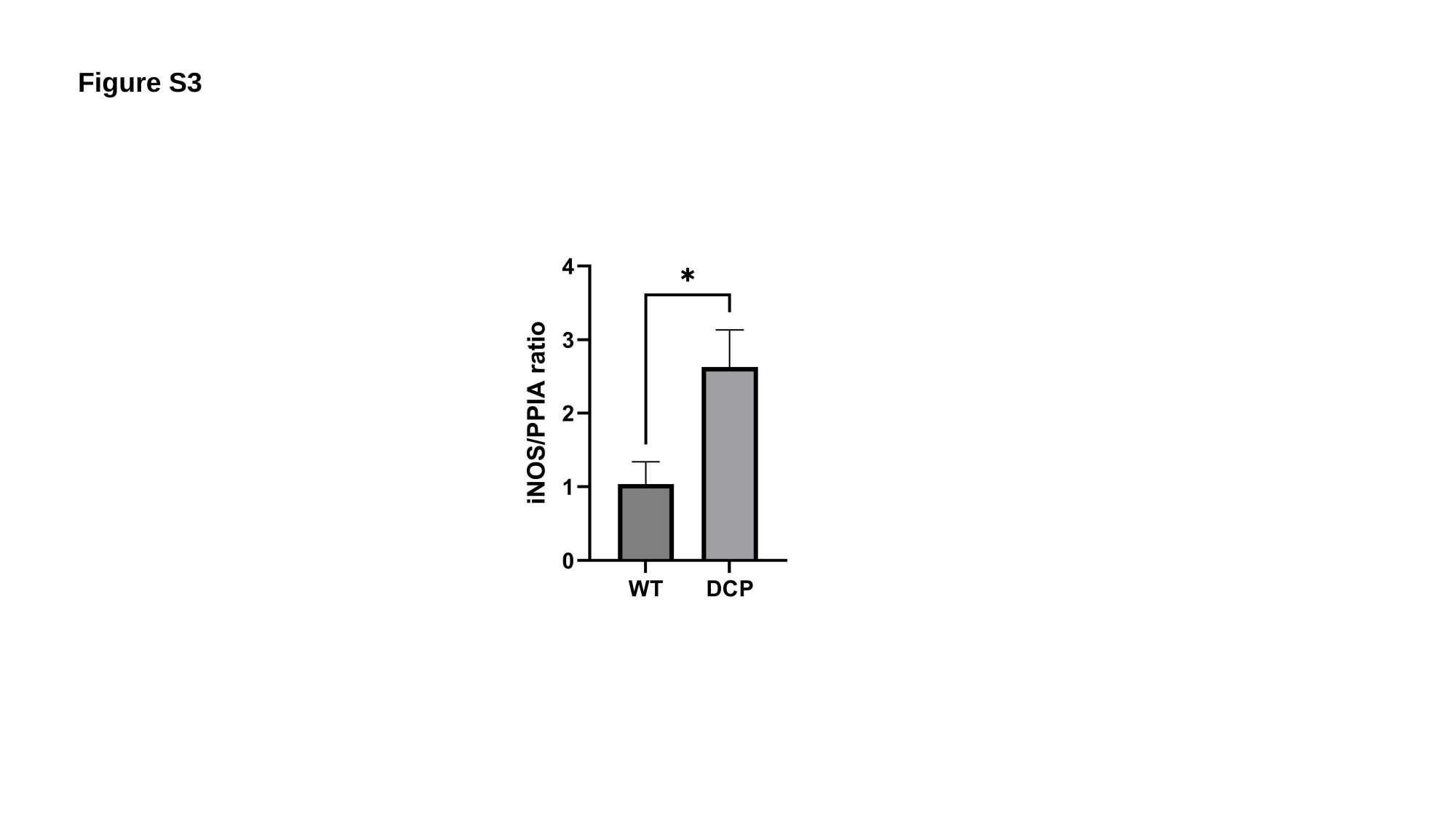

Figure S3
___
